# Acceptability and Feasibility of an Educational Intervention to Improve Researcher-Participant Interactions in a Neonatal Intensive Care Unit Clinical Trial: Research Team Feedback on the BRIEF Intervention

**DOI:** 10.1055/a-2811-5163

**Published:** 2026-02-20

**Authors:** Stephanie A. Kraft, Devan M. Duenas, Andrea Kelsh, Ellie Oslin, Megan M. Gray, Sandra E. Juul, Elliott M. Weiss, the BRIEF-DIVI Collaborative

**Affiliations:** 1 Department of Bioethics and Decision Sciences, Geisinger College of Health Sciences, Danville, Pennsylvania, United States; 2 Treuman Katz Center for Pediatric Bioethics and Palliative Care, Seattle Children’s Research Institute, Seattle, Washington, United States; 3 Division of Neonatology, Department of Pediatrics, University of Washington School of Medicine, Seattle, Washington, United States

**Keywords:** NICU, neonatal, relationship-building, recruitment, informed consent, training, clinical trials, clinical research, communication

## Abstract

**Objective:**

Interactions with families are essential to successful recruitment conversations that promote informed decision-making about clinical research enrollment. However, there is little evidence about how to implement communication-oriented recruitment training among pediatric clinical research teams. Our objective was to evaluate the feasibility and acceptability of Better Research Interactions for Every Family (BRIEF), a multipart educational intervention to improve relationship-based conversations about clinical trial enrollment with families in the neonatal setting.

**Study Design:**

We piloted BRIEF in partnership with a neonatal clinical research team. Research team members completed surveys following the BRIEF intervention’s online module and the BRIEF group training session. They completed self-assessments after consent discussions before and after the BRIEF intervention, in which they rated their achievement of recruitment skills taught in BRIEF. Research team members also completed a final study interview to provide feedback on the intervention components, training content, and use of skills in practice.

**Results:**

All nine research team members completed all components of BRIEF. Survey responses showed moderate to low satisfaction with previous recruitment training before BRIEF and high satisfaction with the BRIEF training. Self-assessments showed significant increases in reported partnership with bedside nursing (*p* = 0.02) and confirmation of family names (*p* = 0.05) after BRIEF training. Interviews provided further evidence of overall satisfaction with the BRIEF training, its content, and the skills learned, as well as opportunities for improvement, particularly in supporting challenging conversations.

**Conclusion:**

This pilot study demonstrated the feasibility and acceptability of the BRIEF intervention, as well as opportunities for improvement in future training.

## Introduction

Relationship-building is essential to clinical research recruitment and consent. Interpersonal interactions with researchers shape participants’ experiences of respect^1,2^ and can facilitate the decision-making process.^3^ These conversations can be challenging, especially when recruitment takes place within a stressful clinical context where prospective participants and their families may be navigating a variety of medical, social, and financial factors.^4,5^ Clinical trials in the neonatal intensive care unit (NICU) are a prime example of this complexity; in prior work, NICU families have reported feeling overwhelmed with the decision about enrolling in research.^6–8^ Adding to this already-challenging context for decision-making, there may be additional layers of complexity for families who face systematic barriers to care,^9^ which may contribute to nonrepresentative enrollment in NICU clinical trials.^10,11^

Research workforce members who engage with prospective participants need to be prepared to approach families with attention to their unique context. Tailored, relationship-focused approaches can support positive participant experiences and promote shared decision-making.^12^ However, existing research workforce training is inconsistent. A 2015 systematic review on the impact of researcher training programs. primarily in oncology, indicated an increase in researcher confidence in participant communication but minimal impact on the participant side.^13^ A more recent qualitative assessment, also in oncology, identified a need for a focus on inclusive and culturally responsive recruitment.^14^ One recent study in the neonatal setting examined the acceptability of a training session coproduced with clinician, researcher, and family input. The 2-hour training. which included a focus on communication skills and rapport building, was viewed as generally acceptable but was only attended by 4 of 11 researchers who had registered.^15^ Another intervention implemented across five trials, all with adult participants in either oncology or surgical settings, showed promise to support enrollment, improve communication issues during recruitment, and change researcher practice.^16^ These examples illustrate the need for rigorously developed training that can feasibly be implemented among neonatal research teams.

In this paper, we describe the acceptability and feasibility of a novel, rigorously developed educational intervention aimed at strengthening researchers’ relationship-building skills during recruitment and consent for neonatal clinical trials. The intervention development process ^17^ and the impact of the intervention on families’ experiences^18^ have been previously published. Here, we report on the experiences of researchers who underwent the Better Research Interactions for Every Family (BRIEF) intervention training during a pilot study in partnership with a neonatal clinical trial, with the goal of examining the implementation of the educational intervention among a neonatal research team.

## Materials and Methods

### Overview

**Fig. 1:**
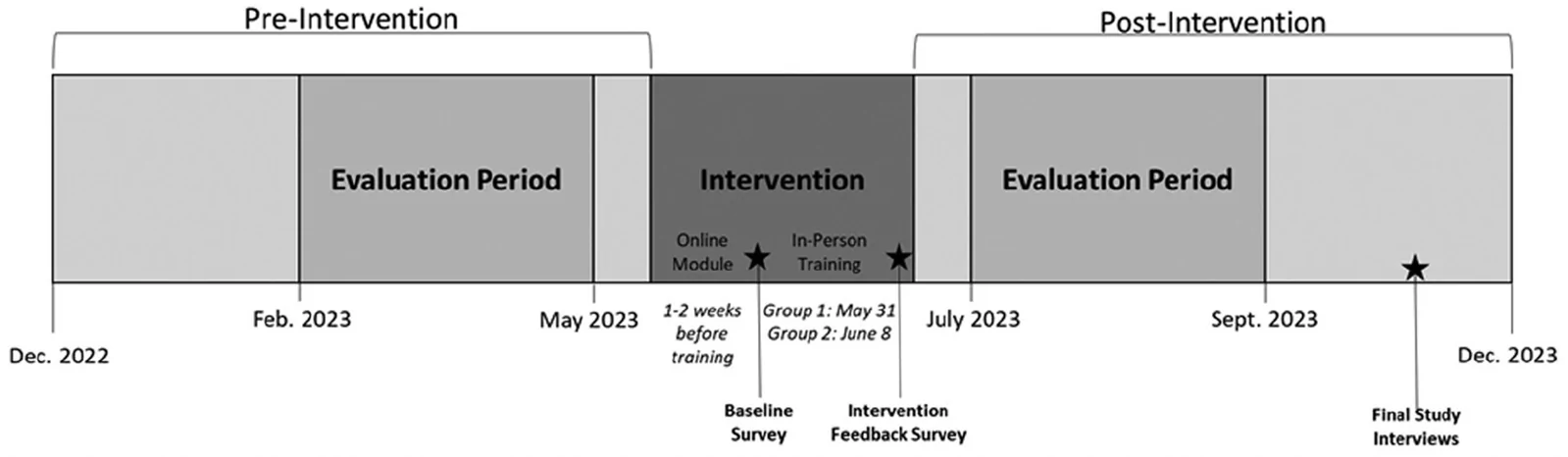
BRIEF intervention pilot study timeline. BRIEF, Better Research Interactions for Every Family.

The BRIEF intervention is a novel, researcher-facing educational intervention developed for use during recruitment approaches for neonatal clinical trials that aims to (1) improve families’ experiences with recruitment and (2) reduce disparities in enrollment. The intervention development process, which used the rigorous, multistep intervention mapping framework,^19^ is described in detail in a prior publication.^17^ BRIEF teaches skills to accomplish 10 performance objectives, based on prior literature and feedback from NICU families, to support researcher-parent relationship-building. Performance objectives were divided among four stages of the recruitment approach: Pre-Approach, Initial Connection, Building Connection, and Follow-Up. Fig. 1 shows our pilot study timeline.

We piloted the BRIEF intervention as an ancillary study to the Darbe plus IV Iron (DIVI) trial, a single-site phase II randomized controlled trial that aims to demonstrate the feasibility and possible benefit for darbepoetin plus slow-release intravenous iron to decrease transfusions, maintain iron sufficiency, and improve neurodevelopmental outcomes of preterm infants.^20^ We invited all DIVI study team members who could be involved in recruitment and consent to participate in the BRIEF intervention. This ancillary study was approved by the University of Washington Institutional Review Board.

### BRIEF Intervention

The intervention, which used a flipped classroom approach, included a 35-minute asynchronous online preclass module and a 2-hour in-person session. The online module included two videos produced by the BRIEF team, in collaboration with the Seattle Children’s Marketing and Communications team, showing short didactic presentations, NICU parent interviews, and example skits illustrating specific skills. The first video provided an overview of relationship-building in research, and the second described and modeled the performance objectives. Short self-reflection questions followed each video.

For this pilot study, we held two in-person group training sessions with the same content, on May 31 and June 8, 2023. The training included a short review of the video content and reflection questions, a didactic presentation of the performance objectives (20 minutes), and four role-play scenarios with standardized parent actors (70 minutes). The role-plays began with a simulation learning contract to foster full participation in the exercise, followed by small group case-based practice discussions. We developed two scenarios that each targeted 2 to 3 of the 10 performance objectives. DIVI team members were asked to incorporate these performance objectives into a mock discussion with the standardized parent actor. One scenario reflected the DIVI trial approach, and the second described a hypothetical randomized controlled trial comparing two standard weaning practices for noninvasive respiratory support in extremely preterm babies. The full case studies are available in a prior publication.^17^ The training session concluded with group reflections and discussion.

### Measures

We collected baseline demographics, including prior recruitment experience, at the end of the online module, and feedback on the intervention via a survey immediately after the group training. Data were collected in a 3-month period before BRIEF and compared with the 3 months’ postintervention: for each period, DIVI research team members were asked to complete a self-assessment via a REDCap survey after each consent discussion with a family. The self-assessment asked them to rate their achievement of each performance objective during the conversation on a scale from 1 to 5, using a team-developed rubric with descriptions and examples of each level, which has been published elsewhere.^17^ We also asked DIVI team members to complete a final interview as BRIEF activities concluded in November 2023, approximately 6 months after the training. Interviews used a descriptive qualitative approach^21^ to seek feedback on the intervention and performance objectives and experiences implementing the BRIEF skills. See Supplemental Material (available in online version only) for interview questions. Interviews were conducted and recorded over Microsoft Teams and lasted approximately 30 minutes. DIVI team members were offered a total of $200 for their participation over the approximately 12-month project period, which included completing the online module, group training, self-assessments during the evaluation periods, and final interview.

### Analysis

Survey data were collected using REDCap and analyzed with STATA^22^ and are presented using descriptive statistics. For self-assessment data, median scores for each performance objective were calculated across all responses and compared pre- versus postintervention using the Wilcoxon rank sum test. Interview recordings were professionally transcribed, cleaned of identifying information, and uploaded to the cloud-based application Dedoose^23^ to facilitate data management and analysis, using a descriptive content analysis approach.^24,25^ We developed an initial deductive codebook based on the topic areas we explored in the interview guide. Two team members (A.K. and D.M.D.} piloted and revised the initial codebook based on transcript review. Each transcript was coded by two team members (A.K., D.M.D.), and any discrepancies were resolved through consensus. Coded data were discussed with the study team, which included investigators and research staff in bioethics and neonatology, to identify key outcomes.

## Results

### Participant Characteristics and Prior Recruitment Experience

All nine DIVI research team members participated in all components of BRIEF, representing a 100% participation rate. Team members included one principal investigator, six coinvestigators, and two research coordinators. A majority self-reported white race (89%), non-Hispanic ethnicity (89%), and female gender (56%). Most reported moderate or high baseline comfort and skill with recruitment before BRIEF, but moderate or low satisfaction with prior recruitment training. Table 1 details research team members’ self-reported baseline experience. Due to small sample size, detailed demographic data are not provided.

### Feedback on BRIEF Intervention

Immediately after the training, DIVI team members responded positively to all components and characteristics of BRIEF (Table 2). All either agreed (*n* = 4, 44%) or strongly agreed (*n* = 5, 56%) that the online module content was interesting, engaging, and helped prepare them for the training session. Most strongly agreed (*n* = 7, 78%) that the goals of the BRIEF intervention were clear and all strongly agreed (*n* = 9, 100%) that the intervention met its goals. All DIVI team members either agreed (*n* = 4, 44%) or strongly agreed (*n* = 5, 56%) that BRIEF would change their practice recruiting families in the NICU.

### Pre-Post Intervention Comparison of Self-Assessment Scores

DIVI team members completed self-assessments following 9 consent discussions during the preintervention evaluation period and 13 during the postintervention evaluation period. Scores on two performance objectives significantly increased from pre- to postintervention: Partnership with bedside nursing (*p* = 0.02) and family names (*p* = 0.05). Median scores and p-values are shown in Table 3.

### Post-BRIEF Interviews

In the final study interviews, DIVI team members described their experiences with and feedback on the two components of the BRIEF intervention: (1) the online module and (2) the group training, as well as on (3) the training content, and (4) implementation of the BRIEF skills in practice. Example quotes for the training components are shown in Table 4 and content and implementation in Table 5.

### Asynchronous Online Module: Videos and Reflection Questions

All DIVI team members recalled the videos positively or neutrally and most said the length was “fine,” although some reported limited recall at the time of the interviews (~6 months after they completed the module). Most said they appreciated hearing a diverse range of families’ perspectives in the videos, with some noting this helped them think about connecting with families from different backgrounds. Three said it was helpful to see the steps laid out clearly to communicate with the care team and get to know a family before jumping into a recruitment conversation. One said the realistic interactions shown in the videos were helpful in preparing them for real-life recruitment situations. Recommended changes to the videos included showing a counterexample of a poor approach, hearing from parents who had negative experiences, and navigating difficulties partnering with clinical staff. Two DIVI team members also suggested adding quick-reference tools to review the content later.

Most DIVI team members said the reflection questions were helpful for allowing them to reflect on their previous recruitment interactions and reevaluate those experiences in the context of the new skills they learned in BRIEF. One said the questions led them to recognize the tools and skills they already had. Only one person said they felt the questions were too long. Another said they would have appreciated being able to see the questions before watching the videos to be able to reflect during the videos.

### In-Person Group Training Session

Most DIVI team members reported that working with the standardized parent actors was helpful, saying they added nuance and realism to the scenarios. Several said the role-play scenarios were beneficial for allowing them to practice using the BRIEF skills and better understand what an ideal approach might look like. However, one noted they felt overwhelmed reviewing the detailed rubric of performance objectives while interacting with the parent actors. Two people brought up that the in-person aspect added to the realism of the approach, although one said a virtual training could help prepare for the increasing number of consent conversations occurring remotely. When asked about the possibility of conducting the training remotely, four highlighted the importance of ensuring session logistics run smoothly, and three said it might be harder to engage online with limited nonverbal communication. Some also raised concerns over how to optimize exchange of feedback in a virtual setting.

Feedback on the scenarios centered on improving relevance of the cases: four DIVI team members said they would have liked to see both clinical scenarios as applicable to their study, particularly regarding the age of the infant approached for research, and two said it would be helpful for the recruitment approach in the scenarios to match that of their study. One commented that cases should represent real life as much as possible, including interactions that do not go positively and families who are resistant to discussing research. One physician team member said that they would have appreciated clearer medical details in the scenarios, and one research coordinator team member suggested holding different sessions for research coordinators and physicians to accommodate different learning needs.

### Training Content: Performance Objectives

Three DIVI team members said that they had previously used some of the performance objectives in their work and that not all were new to them; however, one said they had never previously used any. When asked which were the most helpful, six DIVI team members said that making a concerted effort to check about family names was helpful in connecting with families. All the other performance objectives within the Initial Connection section (*Options for discussing research, Empathy with the NICU family experience, Family needs*) were also mentioned. Several DIVI team members also highlighted the Pre-Approach section, noting it was helpful to focus on partnering with the clinical team and bedside nursing. One person said the Building Connection section was helpful, specifically discussing the performance objective *Options for participation.*

When asked about changes or additions to the training content, DIVI team members suggested guidance on specific challenges in the NICU setting: accounting for short recruitment windows and the resulting time pressure on families; coordinating between families, clinical teams, and research teams when families are eligible for multiple studies; navigating prenatal approaches when eligibility is uncertain; and updating families when their babies are no longer eligible. Other suggestions included adding: more examples of communicating about what other families decide regarding participation; chart review as a step in the Pre-Approach section; strategies for discussing the importance of neonatal research; more detail about partnering with nursing; and logistics when families prefer night or weekend discussions.

### Implementation of BRIEF Skills in Practice

When asked to reflect on their implementation of their BRIEF skills, almost all had positive remarks, saying that it felt good or “fairly organic.” Several DIVI team members said they had used the BRIEF skills in other research or clinical interactions outside of the DIVI study. Even those who did not recruit any DIVI participants reported that the training helped them reflect on their values and what they could bring to future recruitment conversations, giving them more confidence when engaging with the clinical team. Those who were actively recruiting reflected on their interactions with families, saying they felt the skills supported better connections, improved their ability to navigate challenging recruitment experiences, made them more conscientious about knowing family members’ names and state of being, and made them more likely to talk about the rationale and potential benefits of the study. Challenges included coordinating with the clinical team and increased time to complete the full process, particularly when considering which members of the clinical team to ask depending on whether it was a prenatal or postnatal approach. One person noted the steps in the Pre-Approach section sometimes took an extra 15 minutes.

Most DIVI team members perceived that their implementation of the BRIEF skills had been received positively by families. One said they felt families appreciated connecting with the researcher and hearing about the team’s investment in the trial. Others felt families seemed more comfortable when the BRIEF skills were used and appreciated when they checked in about family names. DIVI team members identified the performance objectives in the Pre-Approach and Initial Connection sections as most effective, particularly partnering with bedside nursing and confirming family names. They identified the *Ongoing connection* performance objective in the Follow-Up section as harder to implement, for reasons including lack of established connection making follow-ups difficult or challenges breaking from the “cruise control” mode of the study following enrollment. Some also raised challenges with partnering with nursing staff, as an extra step that took additional time. Another said it was difficult to figure out how to appropriately convey to families information about the benefits for future infants.

Some DIVI team members spoke more broadly about challenges they faced with implementing the skills, noting that challenges varied based on families’ needs. One constraint was difficulty finding the right time to discuss the study, given the tight window for recruitment. Another commented that it was difficult to implement the skills when a family declines an approach. One research coordinator shared that it was difficult when the family would ask specific medical questions, as the coordinator would have to defer to the clinical staff, which they felt compromised their credibility with the family. When asked to reflect on approaches that they felt went poorly, several DIVI team members talked about situations where a lack of engagement from one parent or disagreement between parents made the conversation difficult. Another shared an experience when they felt they were unable to connect with family due to the family being “emotionally not in a space” where they were receptive to talking about the study. Two DIVI team members also talked about external factors that inhibited their ability to connect with families, including confusion from multiple studies having approached the family, lack of clarity around whether a discussion is a consent discussion or an introduction, and the infant’s current clinical situation.

## Discussion

All DIVI research team members successfully completed all components of this pilot BRIEF intervention. Overall, they reported positive feedback on the value and impact of each component. Taken together, our findings demonstrate that BRIEF was feasible to implement and acceptable to its target audience, and it can offer a path forward to address inconsistencies in recruitment training for clinical research.

Our findings showed a particularly strong impact on performance objectives within the Pre-Approach and Initial Connection steps of the BRIEF framework. The pre versus postintervention self-assessment data demonstrate that research team members perceived a change in their behavior on at least two of the performance objectives within these steps: *Partnership with nursing* and *Family names.* Interview responses also highlighted these components as particularly novel compared with usual practice and valuable for forming productive relationships with families. These steps toward developing research relationships may help facilitate engagement and support decision-making during informed consent discussions.^26^ These findings demonstrate that our approach changed how researchers prepare for and initiate interactions with families on at least these two performance objectives, both of which occur early in a consent approach and include discrete tasks (i.e., engaging with bedside nursing and confirming how family members want to be addressed). Other objectives may have been tasks researchers already felt they were doing-especially among this relatively experienced group—and our training may have benefited from more concrete strategies for integration throughout a conversation.

Our findings reveal several opportunities for improvement to our approach. First, an important pattern in feedback was that the training would be improved by adding more direct teaching about how to navigate challenging recruitment conversations, for example, when a family is hesitant to talk with the research team or there is disagreement between parents. Specific suggestions included offering sample language for navigating tense situations and demonstrating in the videos how to come back from a poorly received initial approach or misstep in the conversation. Future iterations of BRIEF can build out these components, drawing on clinical communication training curricula that teach skills such as responding to emotions and tailoring information to families’ needs.^27^ Smith and colleagues, in their NICU research recruitment training, similarly emphasize the importance of engaging with families at a difficult and stressful time and spending time to build rapport before discussing research.^15^

A second opportunity for improvement is to tailor future training for different roles. Our findings suggest that individuals in different roles may have distinct strengths and training needs. Some research team members noted differences between physicians and research coordinators, such as clinical knowledge and relationships with others on the care team (e.g., bedside nursing), that may shape their approach to recruitment. Future training could ask participants to reflect on their positionality, unique strengths, and particular difficulties, with an eye toward overall workforce capacity building.

Third, our results highlight considerations for adapting this training for remote delivery. Research team members valued the in-person approach but were open to the possibility of remote training. In fact, several noted this could be preferable if a study were using a primarily remote recruitment approach. Creating modules that are adaptable to in-person or remote approaches will be important for training in the setting of multicenter studies. This can also help improve efficiency of training when working with teams across widespread geographic areas. However, when teams are local and recruitment approaches will take place in person, they may find value in in-person training both for practicing in a realistic setting and for interacting with their colleagues to improve team-based learning and engagement.

This single-site pilot study is limited in its generalizability. While we had a 100% participation rate among the research team, our findings only reflect viewpoints of nine individuals at a single institution; other researchers may find it harder to implement this training in their respective settings. Notably, most of the research team were neonatologists who could draw on their prior clinical communication training to supplement the BRIEF training. These skills may be important to incorporate for audiences of primarily research coordinators who may have less or no experience with clinical communication. Characteristics of the partner DIVI trial may have also impacted our assessment in unknown ways. Additionally, researchers’ positive feedback to BRIEF may stem in part from the team’s prior investment in BRIEF’s goals; developing buy-in with other teams will be an important step in future training implementation. Finally, this paper reports only on researchers’ experiences and perceptions of BRIEF. Family perceptions and enrollment rates are reported elsewhere^18^ and provide important additional context into the impact of this intervention. Nevertheless, our successful implementation of a training intended to promote stronger research relationships is a key first step on the pathway to improving families’ experiences.

## Conclusion

This study demonstrated the feasibility and acceptability of a research team educational intervention to improve relationship-based communication about clinical trial enrollment with families in the NICU. These findings from the research team’s experiences offer support for the intervention’s framework as well as areas for improvement. Future work should build on this pilot study to refine the training approach and assess researcher and family experiences across multiple institutional settings.

## Supplementary Material

Supplementary Material

Supplementary Material is available at https://doi.org/10.1055/a-2811-5163.

## Figures and Tables

**Table 1:** Research team baseline recruitment experience

Characteristics	*N* = 9 (%)
Years employed in current position	
≤10	7 (78)
11–25	1 (11)
>25	1 (11)
Years in a role involving clinical research recruitment	
≤10	6 (67)
11–25	2 (22)
>25	1 (11)
Comfort approaching potential participants for clinical research	
Extremely	2 (22)
Very	1 (11)
Moderately	5 (56)
Slightly	1 (11)
Not at all	–
Skill approaching potential participants for clinical research	
Extremely	–
Very	3 (33)
Moderately	3 (33)
Slightly	3 (33)
Not at all	–
Satisfaction with prior training in clinical research recruitment	
Extremely	–
Very	1 (11)
Moderately	4 (44)
Slightly	2 (22)
Not at all	2 (22)
Experience level with clinical research recruitment	
Expert	3 (33)
Proficient	–
Competent	2 (22)
Advanced beginner	3 (33)
Novice	1 (11)
Number of prior research studies involved in recruitment (not including DIVI)	
1–5	6 (67)
5–10	2 (22)
>10	1 (11)

Abbreviations: BRIEF, Better Research Interactions for Every Family; DIVI, Darbe plus IV Iron.

**Table 2:** Feedback on BRIEF intervention

	Strongly disagree *n* (%)	Disagree *n* (%)	Neither disagree nor agree *n* (%)	Agree *n* (%)	Strongly agree *n* (%)
Prework (videos and questions)…					
…were interesting and engaging	–	–	–	4 (44)	5 (56
…helped prepare me for today’s in-person session	–	–	–	4 (44)	5 (56)
The BRIEF in-person educational intervention…					
…goals were clear	–	–	–	2 (22)	7 (78)
…met its goals	–	–	–	–	9 (100)
…format was appropriate	–	–	–	1 (11)	8 (89)
…team created an atmosphere of psychological safety	–	–	–	1 (11)	8 (89)
…scenarios with Standardized Parents were helpful for me to practice the skills from the BRIEF intervention	–	–	–	2 (22)	7 (78)
…will change my practice recruiting families for neonatal clinical trials	–	–	–	4 (44)	5 (56)
…I would have preferred to have been via videoconferencing instead of in-person	6 (67)	2 (22)	1 (11)	–	–
…length of time was appropriate	–	–	–	2 (22)	7 (78)

Abbreviations: BRIEF, Better Research Interactions for Every Family; DIVI, Darbe plus IV Iron.

**Table 3:** Self-assessment scores by performance objective, before and after BRIEF intervention

	Pre-BRIEF median (IQR) *n* = 9 self-assessments	Post-BRIEF median (IQR) *n* = 13 self-assessments	Pre- vs. post- *p*-value—Wilcoxon rank sum
Preapproach			
1. Partnership with clinical team	3.5 (3, 4)	4 (4, 4)	0.11
2. Partnership with bedside nursing	1 (1, 2)	4 (1, 4)	0.02
Initial connection			
3. Family names	3 (2.5, 4)	4 (3, 4.5)	0.05
4. Options for discussing research	3 (3, 4)	4 (3, 4.5)	0.33
5. Empathy with the NICU family experience	4 (2.5, 4)	3 (2.5, 4)	0.68
6. Family needs	3 (2, 3.5)	3.5 (1, 4)	0.51
Building connection			
7. Research team’s investment in trial	3 (2, 3.5)	4 (2, 4.5)	0.12
8. Benefit for future infants	2.5 (2, 3)	4 (2, 4)	0.17
9. Options for participation	2 (2, 3)	4 (2, 4.5)	0.07
Follow-up			
10. Ongoing connection with family	3 (2, 3)	4 (1.5, 4)	0.30

Abbreviations: BRIEF, Better Research Interactions for Every Family; IQR,interquartile range; NICU, neonatal intensive care unit.

**Table 4:** Example quotes: BRIEF intervention components

Topic	Example quote
**Online module**	
Videos	
Diverse range of families	“Hearing from … diverse families in regarding their experiences about research and what they look for or what they feel lacks in terms of research, I think, was helpful.” (*DIVI Team Member G*)
Illustrated steps to connect	“[The videos showed] some mothers who … were completely overwhelmed and they really appreciated several of the methods that neonatologists helped bridge their experiences and help her feel more welcome and informed about her stay. I thought that was a very positive video and I thought it was great, especially being a nonclinician. We don’t get those interactions kind of on the research side as often as clinicians do so I thought that was really good to have that as a resource.” (*DIVI Team Member C*)
Suggestion to show poor approach	“[You could) share a video of everybody doing things really wrong. I think that you might think you’re doing right in this kind of scenario [but] that could be better, so maybe having one that’s close to good but missing some of those key things that you find in the study to be important.” (*DIVI Team Member H*)
Suggestion to add reviews	“I do think it probably would be helpful to just have quick reviews, like an email reminder of these are the quick steps for the BRIEF training.” (*DIVI Team Member D*)
Reflection questions	
Opportunity to reflect on past experiences	“I think any opportunity to reflect on things that you’ve done in the past is helpful. If you’ve watched a video of what you’re hoping the research coordinator or the person consenting is doing in a positive way, then I guess it’s helpful to reflect on what’s happened in your own experiences.” (*DIVI Team Member H*)
Helped recognize own skills	“I think that’s very meaningful to not only watch one of those videos but make you reflect on your own interactions. Sometimes that makes you discover tools that you didn’t know you have, and sometimes the video makes you think about tools that you can use in different situations” (*DIVI Team Member A*)
Improve connection to videos	“Maybe having some sort of primer before you see the video so that as you’re going through the video, you can make some parallels to your own experience, and so you can probably then pick out things a little bit differently.” (*DIVI Team Member B*)
Training session	
Role plays with standardized parent actors	
Added nuance and realism	“What I liked about these actors is that they pushed you a little bit, but they weren’t over the top. I think that kind of subtlety and nuance is appreciated as more reflective of real life.” (*DIVI Team Member D*)
Benefits of role play practice	“I think that even though we all hate role playing, it’s probably the best way to practice to be honest. It kind of forces you to put on your humble hat and actually think, ‘Oh gosh, that is how I talk to people? Maybe I can phrase things differently or things that I think are pretty simple are still not very simple or I am not doing a good job reading the room quite yet.’” (*DIVI Team Member I*)
Rubric was overwhelming	“I was a little overwhelmed by the rubric that you guys were working off of … That was a little hard to look at in terms of like, ‘How can I make this better?’.” (*DIVI Team Member H*)
In-person vs. remote delivery	
In-person group engagement	“Getting people in the same room when you’re discussing things that are challenging, not necessarily even just the actor and the provider, but having the providers together to have that kind of camaraderie and talk about things, I think it opens it up for a little bit of a more dynamic and a little bit deeper of a conversation.” (*DIVI Team Member B*)
Nonverbal communication	“You just see the face of the person [in a virtual training). You’re potentially missing out on a lot of other body language that might be occurring in the background, and you can’t pick up on that if you can’t see people’s hands or how they’re fidgeting or sitting or acting restless and such.” (*DIVI Team Member E*)
Match training to consent approach	“If the [study consent] approach is online, then training online would be the most beneficial … and maybe today, we are somewhere in the middle where some approaches are virtual and some approaches are in-person. Maybe developing a training for both would be the most beneficial.” (*DIVI Team Member A*)
Scenarios	
Tailor cases to study	“Having to talk to a family about a 7-week-old sort of was totally outside what we would be doing for the DIVI study, at least as the first approach to a family, so that didn’t seem to fit.” (*DIVI Team Member E*)
Include a challenging case	“[I would suggest cases with] families who are just outright not interested. They’re interested in research, and then they shut you down brutally. We’ve all had those interactions, and they are awful. But doing it in front of your peers and basically allowing yourself to just leave and reassure the parents are in good hands, even if they don’t want to participate, I think would also be very good too.” (*DIVI Team Member C*)
Tailor training to role	“Maybe [make it] a little bit different for [] physicians versus research coordinators … [One case] had a lot of medicines, and I just didn’t know how to talk to a family about that.“ (*DIVI Team Member G*)

Abbreviation: DIVI, Darbe plus IV Iron.

**Table 5:** Example quotes: Content and implementation of BRIEF training

Topic	Example quote
**Content of training**	
Helpful aspects of training content	
Improved existing skills	“I think I was [using some of these skills before BRIEF], but I’m doing a little better now in terms of, ’Do you want to meet in your room or in the baby’s room?’ and, ‘What time is good for you?’ and that sort of thing.” (*DIVI Team Member F*)
Emphasis on family names	“Family names, instead of just saying mom. That was a huge thing. Everyone says it. So just asking them what they would like to be called.” (*DIVI Team Member G*)
Value of preapproach	“Framing it in the preapproach was a value-added of this study for me … really investing some time of why we’re doing this, where the parents are at, and when’s a good time to come in, what’s going on, being able to just frame it better was really helpful.” (*DIVI Team Member B*)
Value of initial connection	“I think that the initial connection I thought is like the lead-in to any conversation. I think just having a really good foundation for any conversation with the family is the most important part of the process.” (*DIVI Team Member C*)
Encouraged engagement with families	“When you need to consent for a study, you feel like there’s a time crunch. You’ve just traveled specifically to the unit just to talk to this family about this one thing. And so I think it was helpful to have a reminder of some other things that you could do to make the family feel more comfortable.” (*DIVI Team Member H*)
Challenges and suggestions	
Challenge of time pressure	“I think for some studies the window to approach and consent is extremely short. I think taking into account the time pressure is huge.” (*DIVI Team Member A*)
Multiple studies in NICU	“What happens a lot is in a very busy unit with multiple studies going on, these parents or nursing get confronted by multiple different people at different times. And some of the frustration comes from, ‘I already told this person or yesterday,’ and it’s kind of repeated.” (*DIVI Team Member B*)
Adjusting for prenatal approaches	“I saw a lot of mothers before they delivered, so they didn’t have a NICU family experience, nor did they really have bedside nursing per se because the nurses that were caring for them were the [obstetrics] nurses, and they didn’t know basically anything about this study, but they seemed to be very supportive in us talking with families.” (*DIVI Team Member E*)
Suggestion to add reviewing medical record	“I would also add [reviewing] the medical record. So much happens with the screening and learning about what the particular problems are for the parents, social work notes, problem lists within the [electronic medical record], and other things. I think that that is sometimes overlooked because you’re just looking for gestational age and a particular condition, but you’re also missing that the mother, the family might have had some trouble with the previous pregnancy and they are very anxious.” (*DIVI Team Member C*)
Implementation of BRIEF skills in practice	
Impact of BRIEF skills	
Positive experience using BRIEF skills	“It comes back for me … [to] taking those extra minutes to really know the names of all of the family members, know where they’re at, know where the baby’s at, and making sure that I’ve touched points with all of those kind of key people before going into the room. So that when the family knows that I’m invested in them as individuals and their baby.” (*DIVI Team Member B*)
Used skills in other settings	“I hadn’t [previously] learned tips and tools, how to approach a family and how to approach a clinical team. I hadn’t had that type of training. So this to me was eye-opening. … This is exactly what I needed when I’m consenting in my other study.’” (*DIVI Team Member G*)
Positively received by families	“I think the families appreciate that we’ve learned a little about them either from their doctors or their bedside nurses. If the baby’s already born, they seem to appreciate that we’ve sort of checked in on the child, see how the child is doing in general.” (*DIVI Team Member E*)
Challenges using BRIEF skills	
Requires added time	“There was a lot of running around and then trying to capture the attending to talk with them and then running to the bedside nurse. … I felt like there’s just a lot more legwork.” (*DIVI Team Member H*)
Follow-up is challenging	“The follow-up part, I don’t do a very good job with thinking about once the baby’s enrolled. We kind of do the check mark like the baby’s in, and it’s cruise control.” (*DIVI Team Member B*)
Variation across families	“It’s nice to get some tools and some verbiage on how to react to families in different scenarios. So I think the more you practice either through training or real life, the better you become at responding to the families’ reactions, needs, body language, emotions, answers, consent or not.” (*DIVI Team Member A*)
Families may not be ready	“It’s hard to build connections when there’s not buy-in from everybody in the room, and I think that is something that really deters a positive recruitment experience.” (*DIVI Team Member C*)

Abbreviations: BRIEF, Better Research Interactions for Every Family; DIVI, Darbe plus IV Iron; NICU, neonatal intensive care unit.

## Key Points

- It was feasible to implement the BRIEF researcher training in a single-site NICU trial.
- BRIEF training was acceptable to research team members.
- BRIEF training shows potential to improve relationship-based research communication.
