# Supplementary material for: Acceptability and Feasibility of an Educational Intervention to Improve Researcher–Participant Interactions in a Neonatal Intensive Care Unit Clinical Trial: Research Team Feedback on the BRIEF Intervention
Source: Am J Perinatol. 2026 Mar 11;43(12):1678–87. doi: 10.1055/a-2811-5163 (PMC13055289; doi:10.1055/a-2811-5163)
Supplement: Supplementary file 1 [file 10-1055-a-2811-5163_29438486.pdf]

Supplemental Material: DIVI Team Interview Guide

To get started, I'll ask you to think back to the BRIEF training, including the videos, online surveys, and our in-person training session with standardized parent actors. As you know, our study is a pilot study of the BRIEF intervention, so these next few questions are aiming to understand how we can improve different aspects of our training.

1. First, please think back to the two videos we sent you before the in-person session. As you might recall, the first video talked about the importance of diversity in neonatal research and of forming relationships with families during recruitment. It had several interviews with NICU parents about their experiences forming relationships with clinicians and researchers. The second video shared some specific strategies for building relationships and included some skits showing how these could be used in interactions with families.
    a. What was helpful about the videos?
        - [if they can't remember specifics] It's okay if your comments are more general. For example, were there any overarching takeaway messages that stuck with you?
    b. What changes would you want to see in a future version of the videos?
        - [if not raised] What did you think about the length of the videos?

2. Next, as you may recall there were also some questions on the web platform following each video. These questions asked you to reflect on past recruitment interactions that went well or where you had challenges and to think about how you might use strategies from the videos in future recruitment interactions.
    a. What was helpful about these questions?

- [if they can't remember specifics] It's okay if you don't remember the specific questions. What was your general impression from answering these questions? How did they support your learning?

b. What changes would you make to these in a future version?

- [if needed] For example, would you ask more or fewer questions?

3. Now I'd like to ask some questions about the content of what you learned. I'm going to share my screen and show a list of our ten relationship-building strategies in BRIEF. *[share screen]* As you may recall, these were split across four stages of relationship building: (1) before approaching the family, where we focused on partnering with the clinician and the nurse; (2) while forming an initial connection with the family, where we focused on confirming how families wanted to be addressed, providing options for discussing research, empathizing with the NICU family experience, and checking in on family needs; (3) building a connection with the family, where we focused on conveying the research team's investment in the trial, discussing the benefit for future infants, and presenting options for participation; and (4) following up, where we focused on creating an ongoing connection with the family.

a. Which of these were most helpful?

b. Would you consider changing any of these in the future? Which ones, and how would you change them?

c. What topics are important for neonatal research that we should have included or spent more time on?

d. *[optional prompt if needed]* What parts, if any, were repetitive with things you had previously learned outside of BRIEF?

4. Next let's talk about the in-person session with the standardized parent actors. This was our session with the two parent actors. As you might remember, we went through four example cases where everyone practiced using the strategies we discussed. The first two cases involved an infant who was eligible for DIVI but whose parent was waiting anxiously for an ultrasound result. The second two cases involved a 7-week-old infant who had a complicated stay, with a parent who is dealing with financial challenges and has had prior bad experiences with doctors.
    a. What was most helpful about this training session?
    b. What could be improved about the scenario role plays? *[optional prompt if needed]* What do you think the BRIEF study team should know when they are facilitating these types of interaction scenarios?
    c. There's a possibility that a future version of this project might need to be held remotely. What should the team consider if they use a remote approach?

Thanks for all your feedback on the different parts of the BRIEF study. Now I want to ask a little more about how it's felt using what you learned in BRIEF.

5. How has it felt for you to implement the skills and techniques from the BRIEF training into your practice recruiting families for DIVI?

    Optional prompts to be used if needed:
    a. What skills or techniques have been easier to implement?
    b. What skills or techniques have been harder to implement?

6. How have the skills and techniques from the BRIEF training landed with the families you have talked to?

   Optional prompts to be used if needed:

   a. Which skills or techniques have been most effective in helping you connect with families?
   b. Which skills or techniques have been least effective in helping you connect with families?
   c. Can you think of an example where you effectively used skills from BRIEF to connect with a family?
   d. Can you think of an example where you weren't able to connect with a family using skills from BRIEF?

7. Have you used the skills from BRIEF in other settings?

   Optional prompts to be used if needed:

   a. Other research settings outside of DIVI?
   b. Clinical settings outside of research?

Thank you for all your feedback. We will incorporate your and the rest of the team's feedback into the next steps for the BRIEF intervention.

8. Before we end the interview, what other thoughts or feedback do you have about any part of the BRIEF intervention and our approach to studying it?

Thank you again for your time.
